# Does Depression Impact Cognitive Impairment in Patients with Heart Failure?

**DOI:** 10.1155/2012/524325

**Published:** 2012-08-07

**Authors:** Z. N. Sohani, Z. Samaan

**Affiliations:** ^1^Population Health Research Institute, Hamilton, ON, Canada L8L2X2; ^2^Department of Psychiatry and Behavioural Neurosciences, McMaster University, Hamilton, ON, Canada L8S4L8

## Abstract

Prevalence studies have noted the cooccurrence of cognitive decline and depression in persons with heart failure. Cognitive impairment is associated with significant mortality and deteriorated quality of life, likely due to impairments in memory and executive function, which impact a patient's ability to understand and comply with prescribed treatment plans. This is especially true in complex diseases such as heart failure. Evidence from literature supports the possibility of a pathophysiological relationship between cognitive impairment, depression, and heart failure. Yet, very few studies have sought to investigate this relationship. This paper reviews current literature on the association between depression and cognitive impairment in persons with heart failure and explores possible mechanisms explaining this complex triad.

## 1. Introduction

Heart failure (HF) is a complex clinical syndrome characterized by a combination of symptoms and signs including shortness of breath, fatigue, edema as well as functional and structural cardiac abnormalities [1]. HF is estimated to affect about 3.9% (95% CI 3.0, 4.7) of the population aged 55 years or older [2] and 6.7% (95% CI 5.6, 7.9) of those aged 65–84 years [3]. Other studies have shown HF prevalence to increase with age, from as low as 0.9% of individuals aged less than 65 years up to 17.4% in those aged 85 years and older [4]. The burden of HF will likely continue to increase as the population ages. In fact, HF is a leading cause of hospital readmission and mortality [5]. Though less frequently examined, decline in cognitive functioning is common in patients with HF. A systematic review and meta-analysis including over 700 patients with congestive HF showed a significant reduction in attention and memory scores in patients compared to controls suggesting that cognitive impairment (CogI) may be associated with HF [6]. Further, a systematic review including 2937 patients and 14,848 controls showed a substantial (62%) increased risk of CogI in patients with HF [7].

Research into this cooccurrence has found CogI to impart an increased risk of mortality in those with HF (adjusted relative risk (RR) = 4.9; 95% CI 2.9, 8.3) [8]. To further the burden in HF patients, many researchers have found an increased prevalence of depression in this group. Data pooled from 36 studies showed prevalence rate of depression in HF to vary from 19.3% when depression is defined based on diagnostic interviews to 33.6% for depression based on questionnaires [9]. Despite the different methods of defining depression, at minimum one in five patients with HF is affected with depression. Consistent with these findings, the prevalence of depression in patients with HF and atrial fibrillation using the Beck Depression Inventory (BDI) questionnaire was 32%. In this study depression score significantly predicted subsequent cardiovascular mortality (adjusted hazard ratio (HR) = 1.57; 95% CI 1.20, 2.07) [10].

While a relationship between depression and CogI is recognized [11–13], few studies have directly examined this association in persons with HF. We review current literature on CogI and HF, with a focus on the contribution of depression to CogI, in HF.

## 2. Cognitive Impairment and Heart Failure

### 2.1. The Impact of Cognitive Impairment in HF Patients

CogI is commonly associated with HF, especially at an older age, with significant impact on activities of daily living and worsening HF prognosis [14, 15]. Studies have demonstrated that more than half of the patients with HF showed cognitive decline when tested using the Mini Mental State Examination (MMSE), a commonly used tool to screen for signs of CogI in older adults [16–18].

A systematic review and meta-analysis of 22 studies investigating the association between CogI and HF reported the prevalence of CogI to range from 25% to 74% in patients with HF [7]. The overall meta-analysis results from 2937 HF patients, and 14,848 controls showed a 62% increase in CogI in HF compared to controls (odds ratio (OR) = 1.62; 95% CI 1.48, 1.79; *P* < 0.0001) [7], demonstrating a clear cross-sectional association between CogI and HF. It is important to note that CogI does not necessarily indicate dementia. The Diagnostic and Statistical Manual (DSM) defines dementia as a global impairment in memory, abstract thinking, judgment, and higher executive functions that are often associated with changes in personality and social functioning as well as other psychiatric or psychotic symptoms [19]. Many patients who have CogI do not fulfill the diagnostic criteria for dementia. CogI is relatively common in the elderly, with one study reporting that 17% of individuals over the age of 65 have experienced CogI without dementia [20]. These results hint toward the possibility that CogI and HF in older individuals are a function of older age. However, this is unlikely given the numerous studies that included similar age “non-HF” control groups where the prevalence of CogI was less than that observed for the patients with HF (see previously mentioned). Furthermore, there have been studies on younger patients with HF, which also demonstrated significant CogI. One such study of 62 patients with an average age of 44.7 years (SD = 10.6 years) found patients to be impaired on half of the neuropsychological measures. Patients were most impaired on neuropsychological test aspects of reasoning and concept formation, attention, and psychomotor skills [21]. Nonetheless, the pivotal role of age in increasing risk of CogI in HF should not be denied; in this younger group, older patients performed worse on neurocognitive testing and had a poorer prognosis of HF [21].

As mentioned earlier, CogI is associated with increased mortality and deteriorated quality of life. CogI can lead to poor health related self-care [22], which can contribute to morbidity and mortality in patients with chronic HF. Health related self-care is a complex process that requires individuals to actively engage in monitoring their health and disease status and treatment aspects [22]. The presence of CogI in HF is associated with poor health-related self-care [22], which could compromise a patient's ability to understand and follow treatment plans and may lead to frequent hospitalizations in patients with HF. One study explored the precipitating factors for hospitalization in predominantly African American patients with HF and found poor compliance with the treatment of HF to be a great precipitator for hospitalization (64%) [23]. A systematic review of treatment compliance in patients with HF found the rate of poor compliance with treatment to range from 21% to 64% in different populations. Compliance rates tended to vary with education, social support, and self-confidence to maintain health status [24].

### 2.2. Mechanisms and Risk Factors of Cognitive Impairment in Heart Failure

A number of mechanisms responsible for CogI in HF have been reviewed [17, 25], which highlight the contribution of several risk factors such as stroke, cardioembolic abnormalities, and cerebral hypoperfusion [5]. In the following we discuss key mechanisms proposed to explain the association between CogI and HF.

#### 2.2.1. Brain Structural Changes due to Hypoperfusion or Infarction

A possible mechanism behind the association of CogI and HF is cerebral vascular hypoperfusion that may occur after HF due to ischemia or stroke [17]. However in the presence of pathological phenomenon such as HF, certain physiological processes attempt to maintain the cerebral blood flow (CBF) homeostasis. For example, in HF where there is a reduced cardiac output, the blood is shifted away from skeletal muscles to the brain so that adequate CBF can be maintained [17, 25, 26]. However as cardiac output continues to drop in HF, the ability to maintain CBF can be compromised; a 30% reduction in CBF can lead to symptoms associated with cerebral hypoperfusion [25] that may eventually contribute to CogI. Gruhn and colleagues used Xe-133 inhalation single-photon emission computed tomography to find a 31% reduced CBF in patients with HF compared to controls [26]. Building on the work of Gruhn et al., Alves and colleagues found the specific brain regions affected by reduced CBF in HF. They report regional CBF reductions bilaterally in the precuneus and cuneus regions as well as in the right lateral temporoparietal cortex and posterior cingulate gyrus. Interestingly, this group found CogI (measured using Cambridge Mental Disorders of the Elderly Examination) to be correlated with regional CBF reductions in the posterior cingulate cortex and precuneus region, highlighting reduced CBF in specific cerebral regions as a potential link between HF and CogI [27].

In addition to compensation for lowered cardiac output, cerebrovascular reactivity, the capacity for vasodilation in the brain, is also an important mechanism in maintaining CBF [28]. Cerebrovascular reactivity may be affected in HF as seen in studies investigating patients with HF compared to controls and demonstrating significant impairment in cerebrovascular reactivity in HF patients [28]. This impairment in cerebrovascular reactivity may further contribute to hypoperfusion and CogI in HF.

Cerebral hypoperfusion may also lead to CogI through cerebral white matter degeneration (leukoaraiosis) [29]. Leukoaraiosis results in demyelination, loss of glial cells, axon damage, and spongiosis [29]. The progression of such degenerative changes impacts different aspects of cognitive function including immediate and delayed memory, processing speed, higher executive functions, as well as global cognition [30].

#### 2.2.2. Shared Risk Factors with Cardiovascular Diseases

In addition to specific neuronal mechanisms, it is known that patients with endometabolic disorders, including hypertension, diabetes, and hyperlipidemia, as well as conditions like small vessels disease, are at a higher risk of cardiovascular disease such as stroke. CogI may develop as a result of multiple risk factors and disorders in such individuals [17, 31–35].

#### 2.2.3. Iatrogenic Effects on CogI in HF

Investigations into anticholinergic side effects of medication sometimes prescribed to patients with hypertension and congestive HF have found that some of these therapeutic agents are associated with CogI [36]. A longitudinal study of 13,004 participants assessed the risk of CogI with use of anticholinergic medication. CogI was determined using the MMSE. The study observed a positive dose-response relationship between anticholinergic medication use and MMSE score [36], although the change in MMSE score was relatively small and perhaps the benefits of using such medications outweigh the risks of CogI. Furthermore, medications, such as beta-blockers, prescribed in HF may contribute to neuropsychiatric disorders and worsening CogI [37]. Nonetheless the use of medications in HF and any other disease status is based on risk benefit analysis, and in the case of HF, such medications could be lifesavers, and their use is not only justified but also essential.

Overall our literature search showed that there are multiple and complex factors that may influence cognitive function in HF in addition; because patients who suffer from HF may suffer from other cardiovascular disease that have shared risk factors that can contribute to CogI, it is difficult to discern the underlying pathophysiological relationship uniquely attributed to CogI in HF.

## 3. Depression, Cognitive Impairment, and Heart Failure

Depression is a common psychiatric disorder characterized by the presence of low mood or loss of interests associated with several other features that are present almost daily for at least two weeks and results in impaired function [19]. Depression is prevalent in patients with HF as well as those that are cognitively impaired. This is especially true in chronically ill older patients with HF [38], in whom the prevalence of depression is greater than hospitalized older patients with other non-HF diseases (36.5% versus 17%) [39]. Meta-analysis of 27 studies reported the prevalence of depression in HF patients to range from 9% to 60% depending on the use of “liberal” or “conservative” definitions of depression. A measure was considered “conservative” if it was ascertained through an interview, review of medical charts for a formal diagnosis, or the use of screening questionnaires explicitly looking for moderate-to-severe depression [9]. The study also found that the prevalence of depression was positively correlated with poorer prognosis of HF [9]. Depression is commonly found in persons with CogI. In a systematic review of literature on HF and CogI including 22 studies, depressive symptoms were associated with CogI in 50% of the included studies [7]. Further, studies showed that depression may have a negative impact on neuropsychological tests results [7], providing an impetus for more investigation into the role of depression in HF-associated CogI.

A possible explanation of the role of depression in HF may be evident through structural brain abnormalities reported with depression in patients with HF. Studies using structural brain MRIs have attempted to find an association between structural brain lesions and depression. Greenwald and colleagues (2001) used MRI studies to explore the role of hypertension and cerebral white matter lesions and subcortical hyperintensities in depression. The authors compared depressed individuals (depression was defined based on DSM-III-R criteria) with hypertension to normotensive controls, hypertensive controls, and normotensive depressed patients. The authors found significant differences between depressed individuals with hypertension compared to controls with hypertension (deep white matter lesions rating percent distribution of 30% versus 9%; subcortical grey matter hyperintensity rating percent distribution 10% versus 0%) and depressed individuals with hypertension to depressed without hypertension (deep white matter lesions rating percent distribution of 30% versus 17%; subcortical grey matter hyperintensity percent distribution of 10% versus 0%) [40]. Another study found ischemia as the cause of white matter hyperintensities in all patients with depression [41]. These studies support a big role of cerebrovascular changes in depression that may also lead to CogI in susceptible individuals. These studies however failed to show a causative effect or the direction of association between these structural brain changes and depression.

The impact of depression on HF can also be seen through environmental and behavioral factors. Individuals with depression show poor compliance to healthy behaviours and engage in additional risks, such as smoking, sedentary behaviour, poor diet, and substance abuse, which may lead to increasing the risks of cardiovascular disease, CogI, or worsening existing conditions [9, 42, 43].

Depression in persons with CogI is marked by abnormalities on neuropsychological tests [44] including impairments in memory, attention, and executive function, such as problem solving (for a review see [45]). Patients with major depressive disorder continue to show deficits in attention and executive function even after remission [46]. A longitudinal study of 436 women found depressive symptoms to predict the occurrence of impairment in cognitive testing including episodic, immediate, and delayed memory (measured using Hopkins Verbal Learning Test immediate recall (HVLT-Imm) and delayed recall (HVLT-del)), psychomotor speed (measured using Trail Making Test (TMT)-A), and executive functioning (measured using TMT-B) [47], thus strengthening the evidence for a consistent association between depression and CogI. Additionally, in a study of 14,089 participants, Pullicino and colleagues studied the relationship between HF and CogI. HF was characterized using self-reported orthopnea and paroxysmal nocturnal dyspnea (PND), depression was measured using the Center for Epidemiological Studies-Depression (CES-D-4) scale, and CogI was assessed using a six-item test derived from MMSE [48]. The authors reported that participants with a highly probable diagnosis of HF had a 1.51 (unadjusted OR) (95% CI 1.15, 1.96) times greater chance of having CogI than participants without HF. Similarly, those with depressive symptoms had a 1.66 (CI 1.38, 2.01) times greater likelihood of being cognitively impaired than those without depression [48]. Interestingly, when the correlation between probable HF and CogI was adjusted for comorbidities and depression, the OR became insignificant (1.25; 95% CI 0.94, 1.67). This might be due to a stronger relationship between depression and CogI or simply because HF, CogI, and depression were based on self-reported symptoms and not a clinically determined diagnosis. Such limitations are understandable in the context of large sample size. Nonetheless, this large study provides valuable data on the association between depression, CogI, and HF. Mechanistic and longitudinal studies may assist in unraveling this complex relationship.

To our knowledge, only Garcia and colleagues have explored both depression and CogI in patients with HF [44]. The authors administered ten neuropsychological tests to 116 HF patients to assess global cognitive function, attention and executive function, memory, language, and motor functioning. The Beck Depression Inventory II (BDI-II) was used to measure depression. Approximately one in five of HF patients scored positively on BDI II (a score of 14 or higher) [44]. Depression was found to predict CogI on executive function, memory, language, motor function, and global cognitive functioning following adjustment for sex, hypertension, and cardiac fitness. However, when additional demographic variables were added to the model, the association between depression and global cognitive functioning became insignificant. The added demographic variables in the full model were not explained [44].

**Figure 1:**
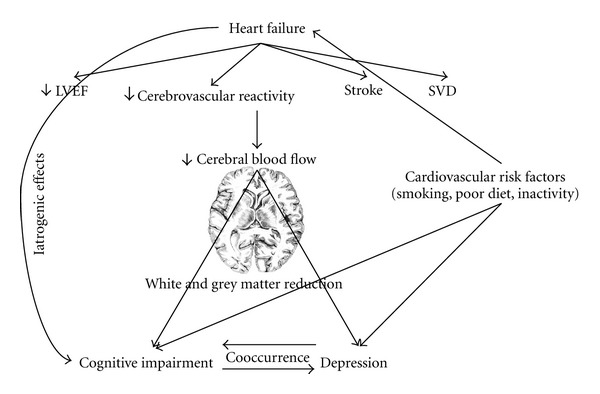
Relationship between CogI, depression, and HF. LVEF2: left ventricular ejection fraction; SVD: small vessels disease.

Overall, the current literature supports the presence of an association among depression, cognitive dysfunction, and HF. In the next section we will explain potential mechanisms behind the association among depression, CogI, and HF.  Figure 1 presents a schematic demonstration of the relationships discussed thus far between the three conditions.

### 3.1. Neural Mechanisms

With the use of neuroimaging techniques, specific brain regions have been investigated for their role in depression. Evidence from literature suggests that depression is associated with an increased occurrence of white matter hyperintensities (WHI) [45]. Sheline and colleagues used MRIs to explore brain regions with WHI in depressed patients. Compared to controls, the depressed group had a greater occurrence of WHI in the right and left superior longitudinal fasciculi, the frontooccipital fasciculus, and the left uncinate fasciculus [49]. The group also found both white and grey matter volume to be correlated with some form of CogI [49]. Other studies have also reported an association between hippocampal volume and CogI in individuals with depression [50].

These findings of cerebral structural changes in depression and CogI, taken with evidence that HF contributes to white matter hyperintensities and whole brain grey matter reductions in cortical and subcortical regions [51–53], suggest that both depression and CogI are a result of underlying structural brain changes caused by HF. However it is not clear from these studies whether depression or CogI predated HF given the shared mechanisms and risk factors. The question arises: does depression cause CogI in patients with vascular pathologies in general or worsen existing CogI as a result of HF? At this point, it is difficult to answer these questions since we do not yet have enough mechanistic or longitudinal studies to ascertain a cause and effect relationship. Additionally, as illustrated in the diagram and the plethora of potential mechanisms described, there can be several pathways underlying depression and CogI observed in patients with HF, and discerning a cause and effect relationship in the context of the multiplicity of pathways is akin to “finding a needle in haystack.” In order to answer these questions, future studies should longitudinally compare persons with HF and baseline depression to HF patients without depression while controlling for other vascular, endometabolic, and inflammatory pathologies. Despite the need for such studies, they may pose feasibility challenges such as sample size given the need to adjust for multiple factors, duration of followup, attrition rate, and cost of conducting such studies. A key challenge in current studies is the use of various tools and definitions for the diagnosis of depression and CogI, which limits comparability between studies. This study of heterogeneity also makes performing well-conducted systematic review and meta-analysis exceptionally challenging.

### 3.2. Neurohormones

Hormones such as cortisol are known to be associated with depression and cognitive function. Elevated cortisol level was reported in current and remitted depression [54, 55] as well as nonsuppression by dexamethasone using dexamethasone suppression tests (DSTs) (for a review see [56]). Prolonged exposure to elevated cortisol levels has shown to reduce hippocampal volume in depression, which has adverse effects on verbal memory [57–59]. Although this relationship is inconsistently reported [60], a randomized controlled trial of high dose cortisol (160 mg/day) compared to low dose (40 mg/day) given to subjects for 4 days demonstrated a negative impact on verbal memory in the high cortisol dose group that was reversible [61]. Similarly, Kirschbaum and colleagues conducted two studies to investigate the relationship between cortisol and cognition. In the first study, participants were asked to take the Trier Social Stress Test. They were then given declarative memory tasks. They were also sampled for saliva to ascertain cortisol levels. The first study found a negative relationship between stress-induced cortisol level and declarative memory. In the second study, participants were assigned to a placebo group or cortisol group. The cortisol group showed impaired performance in the declarative memory and spatial thinking tasks compared to the placebo group [62].

The effect of cortisol may also be important in HF. One study measured serum levels of cortisol and aldosterone in 294 patients with chronic HF [63] and found levels of cortisol to be elevated in HF. In addition, the authors used regression analysis to determine that cortisol and aldosterone predict increased mortality in HF (HR of highest versus lowest tertile for cortisol was 2.72 and aldosterone was 2.19). Additionally, patients with elevated cortisol and aldosterone had approximately a threefold greater risk of dying compared to patients who had median levels of both hormones. Another study, by Yamaji and colleagues, found serum cortisol to predict cardiac events in those with HF [64]. These studies although performed in HF or depression only samples may help to explain the role of cortisol and hypothalamic pituitary axis in the complex triad of depression, CogI, and HF with cortisol impacting each of these conditions. Other hormones such as atrial natriuretic peptide, adrenaline, noradrenaline, and thyroid hormone have a role in HF [65–70], and this role may extend to depression and CogI [71–74]; however these additional mechanisms are beyond the scope of this paper.

### 3.3. Inflammation

Emerging research in the field of inflammation and chronic diseases has shed light on the important role of cytokines in the pathogenesis of HF, depression, and CogI [75]. Elevated levels of proinflammatory cytokines, specifically IL-6, TNF-*α*, as well as C-reactive protein, seem to have a broad role in HF, depression, and CogI. Kubota and colleagues found elevated TNF-*α* and IL-6 in people with HF [76], while other studies reported similar findings in patients with depression and HF [77]. These elevated levels could relate to the HF process rather than depression in this study. But this concern was clarified by further studies of these inflammatory markers where these markers were raised in those with depression and HF compared to nondepressed with HF [78]. Similar findings have been reported in CogI [51–53]. Interestingly, one of the studies delved a bit into analysis of cytokines by sex and found that males with mild CogI had elevated serum amyloid A and C-reactive protein, while females had elevated TNF-*α* [79]. The role of sex is an additional factor which warrants further study in the context of depression, CogI, and HF.

Inflammatory cytokines and their role in depression and heart disease have been thoroughly reviewed [75, 80, 81]. It should be noted that depression is associated with dysregulation of immune response. A study of cellular immune activity in patients with congestive HF and depression (established using the Hamilton Rating Scale) reported that a lower IFN-gamma/IL-10 ratio was related to higher depressive symptoms. Interestingly, no difference in plasma IL-6 levels between those with high versus low depression scores [82] was seen in this study. These findings add further to the complexity of association between inflammatory markers and depression and HF.

A plausible explanation of the effect of proinflammatory cytokines in depression has been provided [83]. Briefly, increased levels of proinflammatory cytokines may reduce tryptophan, an important precursor for neurotransmitters such as serotonin with an essential role in depression. In addition, these cytokines may also affect hypothalamic-pituitary axis (HPA) leading to dysregulation of cortisol response known to impact depression, cognition, and HF as described earlier [83].

While it is interesting that the same cytokines are elevated in depression, CogI, and HF, this commonality does not confer a causative relationship. Depression in HF is associated with increased mortality (OR at 3 months = 2.5, OR at 1 year = 2.23 [84]) which could be due to the harmful effects of cytokines on the heart. Pasic, Levy, and Sullivan in their review propose that cytokines, such as TNF-*α*, mediate sepsis-induced alterations which ultimately reduce contractility and promote left-ventricular dysfunction among other adverse outcomes [75]. Reduced contractility and worsened HF could lead to poor cerebral perfusion ultimately causing CogI. However, the opposite may also be true: cytokines elevated as a result of HF could play some role in the development of depression or CogI or both. Most studies reviewed to date have evaluated a binary association between depression and HF or between CogI and HF or depression and CogI. Future initiatives should seek to prospectively ascertain whether there is a causal pathway between the three conditions.

## 4. Treatment

The association between CogI and depression in HF is complicated, and the conditions can cooccur through many pathways. In the context of HF, hypoperfusion can cause structural changes in the brain which can lead to depression or CogI or both. Or elevated cytokines can decrease serotonin production resulting in depression and disrupt HPA regulation leading to CogI. Sometimes the HPA axis is activated in tandem with the immune system; therefore a combination of these pathways or existence of multiple pathways can also occur. If depression affects an individual's risk of either becoming cognitively impaired or worsens existing cognitive dysfunction, treatment for depression should alleviate some burden of CogI. This theory was tested in a trial of 42 patients with current depression diagnosed according to the criteria of DSM-IV-TR [85]. While some improvement in CogI did occur with antidepressant therapy, the depressed group continued to perform poorly on tests of complex tasks which required problem solving and strategic thinking [85]. Additionally, treating for depression is associated with a high rate of relapse in the presence of CogI [45]. The relapse could result from an inability to comply with depression treatment. There has been some research into whether treatments for HF improve CogI. Studies looking at heart and pacemaker transplantation to improve cardiac function have yielded inconclusive results [21, 86–88]. The discouraging results from attempts to treat CogI and depression in HF are indicative of a failure to understand all the possible interactions and should provide an impetus to continue work in this field to truly discern the cause and effect relationships.

## 5. Future Directions

A key challenge confronting investigators is to discern the causal mechanisms underlying the relationship between cognition, depression, and HF. What has made this endeavor particularly difficult is the possibility of several pathways that affect cognition. To make matters even more complicated, there are factors such as cognitive reserve that make CogI more easily apparent in some compared to others. Brain reserve capacity is the ability to tolerate greater cell loss due to the existence of neuronal redundancy [42]. These individuals take longer than those without such neuronal redundancy to exhibit clinical symptoms. This paper has highlighted several key questions: does depression worsen existing CogI in those with HF? Can treatment for depression change the onset of CogI in this group, and what mechanisms lead to the coexistence of these three conditions? It is very important that in the clinical care of HF patients, medical professionals be aware of the high rate of cooccurrence between HF, CogI, and depression. Health professionals treating for HF should screen for cognitive function as well as depression. Future studies should also seek to investigate neuropsychiatric rehabilitation for CogI and depression in patients with HF in improving heart function as well as reducing rates of hospitalization and mortality from HF.
